# Supplementary material for: The mediating role of depression on the link between physical activity and health-related quality of life among people with diabetes: A cross-sectional study
Source: PLoS One. 2024 Dec 5;19(12):e0312774. doi: 10.1371/journal.pone.0312774 (PMC11620351; doi:10.1371/journal.pone.0312774)

PLOS ONE

The mediating role of depression on the link between physical activity and health-related quality of life among people with diabetes: A cross-sectional study

--Manuscript Draft--

**Manuscript Number:**	PONE-D-24-24159
**Article Type:**	Research Article
**Full Title:**	The mediating role of depression on the link between physical activity and health-related quality of life among people with diabetes: A cross-sectional study
**Short Title:**	The mediating role of depression on the link between physical activity and health-related quality of life
**Corresponding Author:**	Sanghee Kim, Ph.D.<br>Keimyung University<br>Daegu, KOREA, REPUBLIC OF
**Keywords:**	physical activity; depression; mediating role; health-related quality of life; diabetes
**Abstract:**	Introduction<br><br>A correlation between decreased health-related quality of life (HRQoL) and physical activity (PA) has been identified. Many studies have discussed whether this correlation is significantly associated with depression in the general and diabetic populations. However, the role of depression in this relationship, especially in individuals with diabetes, remains incompletely understood.<br><br>Objective<br><br>This study investigated the relationship between PA and HRQoL, with depression as a potential mediator, in individuals with diabetes.<br><br>Methods<br><br>This cross-sectional study involved 1,472 individuals with diabetes who participated in the Korea Health Panel Survey (KHPS) from 2019 to 2020. Their sociodemographic characteristics, PA, depressive symptoms, and HRQoL based on EuroQol-five-dimension (EQ-5D) scores were extracted from the KHPS. The mediating effect of depression on PA and HRQoL was investigated using multiple regression and a mediation effect test.<br><br>Results<br><br>HRQoL was positively associated with PA, regular exercise, and varying degrees of walking activity. Conversely, depression was substantially negatively associated with HRQoL. Mediation analysis confirmed that depression partially mediated the relationship between PA and HRQoL. Specifically, for PA and regular exercise, the indirect effect of depression accounted for 46.61% ($B = 0.002$, $p < 0.05$) and 33.82% ($B = 0.003$, $p < 0.001$).<br><br>Conclusion<br><br>In individuals with diabetes, depression was found to moderate the effect of PA on HRQoL. Therefore, conducting depression screening and managing depressive symptoms in this population is crucial to enhancing HRQoL through PA interventions. Consequently, strategies to enhance HRQoL can be effectively implemented and customized in response to particular depression screening outcomes.
**Order of Authors:**	Djoko Priyono, MSN, RN
	Sanghee Kim, Ph.D.
**Additional Information:**	

Powered by Editorial Manager® and ProduXion Manager® from Aries Systems Corporation

Question	Response
**Financial Disclosure**<br><br>Enter a financial disclosure statement that describes the sources of funding for the work included in this submission. Review the submission guidelines for detailed requirements. View published research articles from *PLOS ONE* for specific examples.<br><br>This statement is required for submission and **will appear in the published article** if the submission is accepted. Please make sure it is accurate.<br><br>**Funded studies**<br>Enter a statement with the following details:<br>• Initials of the authors who received each award<br>• Grant numbers awarded to each author<br>• The full name of each funder<br>• URL of each funder website<br>• Did the sponsors or funders play any role in the study design, data collection and analysis, decision to publish, or preparation of the manuscript?<br><br>Did you receive funding for this work?	The author(s) received no specific funding for this work.
**Competing Interests**<br><br>Use the instructions below to enter a competing interest statement for this submission. On behalf of all authors, disclose any competing interests that could be perceived to bias this work—acknowledging all financial support and any other relevant financial or non-financial competing interests.<br><br>This statement is required for submission and **will appear in the published article** if the submission is accepted. Please make sure it is accurate and that any funding sources listed in your Funding Information later in the submission form are also	The authors have declared that no competing interests exist.

declared in your Financial Disclosure statement.<br><br>View published research articles from *PLOS ONE* for specific examples.<br><br>**NO authors have competing interests**<br><br>Enter: *The authors have declared that no competing interests exist.*<br><br>**Authors with competing interests**<br><br>Enter competing interest details beginning with this statement:<br><br>*I have read the journal's policy and the authors of this manuscript have the following competing interests: [insert competing interests here]*<br><br>* typeset	
**Ethics Statement**<br><br>Enter an ethics statement for this submission. This statement is required if the study involved:<br><br>• Human participants<br>• Human specimens or tissue<br>• Vertebrate animals or cephalopods<br>• Vertebrate embryos or tissues<br>• Field research<br><br>Write "N/A" if the submission does not require an ethics statement.<br><br>General guidance is provided below. Consult the submission guidelines for detailed instructions. **Make sure that all information entered here is included in the Methods section of the manuscript.**	N/A

Powered by Editorial Manager® and ProduXion Manager® from Aries Systems Corporation

**Format for specific study types**<br><br>**Human Subject Research (involving human participants and/or tissue)**<br>• Give the name of the institutional review board or ethics committee that approved the study<br>• Include the approval number and/or a statement indicating approval of this research<br>• Indicate the form of consent obtained (written/oral) or the reason that consent was not obtained (e.g. the data were analyzed anonymously)<br><br>**Animal Research (involving vertebrate animals, embryos or tissues)**<br>• Provide the name of the Institutional Animal Care and Use Committee (IACUC) or other relevant ethics board that reviewed the study protocol, and indicate whether they approved this research or granted a formal waiver of ethical approval<br>• Include an approval number if one was obtained<br>• If the study involved *non-human primates*, add *additional details* about animal welfare and steps taken to ameliorate suffering<br>• If anesthesia, euthanasia, or any kind of animal sacrifice is part of the study, include briefly which substances and/or methods were applied<br><br>**Field Research**<br><br>Include the following details if this study involves the collection of plant, animal, or other materials from a natural setting:<br>• Field permit number<br>• Name of the institution or relevant body that granted permission	
**Data Availability**<br><br>Authors are required to make all data underlying the findings described fully available, without restriction, and from the time of publication. PLOS allows rare exceptions to address legal and ethical concerns. See the PLOS Data Policy and FAQ for detailed information.	No - some restrictions will apply

A Data Availability Statement describing where the data can be found is required at submission. Your answers to this question constitute the Data Availability Statement and **will be published in the article**, if accepted.<br><br>**Important:** Stating 'data available on request from the author' is not sufficient. If your data are only available upon request, select 'No' for the first question and explain your exceptional situation in the text box.<br><br>Do the authors confirm that all data underlying the findings described in their manuscript are fully available without restriction?	
**Describe where the data may be found in full sentences. If you are copying our sample text, replace any instances of XXX with the appropriate details.**<br><br>• If the data are **held or will be held in a public repository**, include URLs, accession numbers or DOIs. If this information will only be available after acceptance, indicate this by ticking the box below. For example: *All XXX files are available from the XXX database (accession number(s) XXX, XXX.).*<br>• If the data are all contained **within the manuscript and/or Supporting Information files**, enter the following: *All relevant data are within the manuscript and its Supporting Information files.*<br>• If neither of these applies but you are able to provide **details of access elsewhere**, with or without limitations, please do so. For example:<br><br>*Data cannot be shared publicly because of [XXX]. Data are available from the XXX Institutional Data Access / Ethics Committee (contact via XXX) for researchers who meet the criteria for access to confidential data.*<br><br>*The data underlying the results presented in the study are available from (include the name of the third party*	This study obtained data from the 2020 KHPS dataset. The KHPS was undertaken collaboratively by the National Health Insurance Corporation and the Korea Institute for Health and Social Affairs. It is a nationwide public database (https://www.khp.re.kr) comprised of households and their members.

Powered by Editorial Manager® and ProduXion Manager® from Aries Systems Corporation

and contact information or URL).<br>• This text is appropriate if the data are owned by a third party and authors do not have permission to share the data.<br><br>* typeset	
Additional data availability information:	

Manuscript

Click here to access/download;Manuscript;Manuscript PLoS One (Revision).docx

The mediating role of depression on the link between physical activity and health-related quality of life among people with diabetes: A cross-sectional study

Short tittle: Depression Mediates Physical Activity and QoL in Diabetes

Abstract

Introduction

A correlation between decreased health-related quality of life (HRQoL) and physical activity (PA) has been identified. Many studies have discussed whether this correlation is significantly associated with depression in the general and diabetic populations. However, the role of depression in this relationship, especially in individuals with diabetes, remains incompletely understood.

Objective

This study investigated the relationship between PA and HRQoL, with depression as a potential mediator, in individuals with diabetes.

Methods

This cross-sectional study involved 1,472 individuals with diabetes who participated in the Korea Health Panel Survey (KHPS) from 2019 to 2020. Their sociodemographic characteristics, PA, depressive symptoms, and HRQoL based on EuroQol-five-dimension (EQ-5D) scores were extracted from the KHPS. The mediating effect of depression on PA and HRQoL was investigated using multiple regression and a mediation effect test.

Results

HRQoL was positively associated with PA, regular exercise, and varying degrees of walking activity. Conversely, depression was substantially negatively associated with HRQoL. Mediation analysis confirmed that depression partially mediated the relationship between PA and HRQoL. Specifically, for PA and regular exercise, the indirect effect of depression accounted for 46.61% ($B = 0.002$, $p < 0.05$) and 33.82% ($B = 0.003$, $p < 0.001$).

Conclusion

In individuals with diabetes, depression was found to moderate the effect of PA on HRQoL. Therefore, conducting depression screening and managing depressive symptoms in this population is crucial to enhancing HRQoL through PA interventions. Consequently, strategies to enhance HRQoL can be effectively implemented and customized in response to particular depression screening outcomes.

Keyword: Physical activity, depression, mediating role, health-related quality of life, diabetes

Introduction

Regular physical activity (PA) is crucial for those with diabetes. It is widely acknowledged to aid in managing diabetes by enhancing glycemic control, mitigating the risks of diabetes-related complications, and promoting overall well-being, encompassing both mental and physical aspects [1,2]. However, compared to the general population, those with diabetes tend to engage in less PA. Furthermore, 40% of those with diabetes do not participate in sufficient physical activity based on the World Health Organization recommendations [3]. One study using the UK Biobank, which comprises over 233,000 participants, revealed that those with cardiometabolic disorders, including

type 2 diabetes, reported significantly less PA, particularly vigorous PA, than healthy adults. This study also reported that 16% of the no-disease group had low PA levels, which increased with the onset of cardiometabolic disease. A lack of PA has also been linked to several risk factors, including multiple comorbidities, being overweight or obese, high stress, and lower health-related quality of life (HRQoL) [4,5].

HRQoL refers to the impact of diabetes and its management on an individual's overall well-being, including physical, mental, and emotional health [6]. While several studies have examined the relationship between PA and HRQoL in the general population, few have focused on individuals with chronic diseases such as diabetes. Therefore, further research in this area is needed [7]. Furthermore, the sample sizes of many studies on individuals with type 2 diabetes have been relatively small, limiting the statistical power and generalizability of their findings. Furthermore, studies have not consistently accounted for important confounding variables, such as diabetes complications, comorbidities, and socioeconomic status, which may independently influence PA and HRQoL [8,9]. Lastly, there is a lack of research investigating potential mediators, such as depression, with individuals with diabetes two- to three-fold more likely to experience depression.

A more comprehensive examination of the processes involved in the relationship between independent and dependent variables can lead to new insights into the timing and nature of their influence. In this study, we considered depression as a mediator in the relationship between PA and HRQoL. Research has revealed that moderate PA levels and leisure activities are related to depression in patients with diabetes [10]. A previous longitudinal study found that moderate-intensity PA was associated with a 28% lower risk of developing major depression [11]. Conversely, depression negatively impacted HRQoL in patients with diabetes [12]. Several studies have shown depression to have a partial mediating effect on the relationships between HRQoL and

neuropathic pain, atrial fibrillation, and cardiovascular disease. However, further investigation is needed in the case of patients with diabetes.

Based on our analysis, we propose possible mediating effects of depression on the relationship between PA and HRQoL. It is crucial to investigate the association between PA and HRQoL and the potential impact of depression on this relationship using data-driven inferences. This study had two primary aims: (i) to assess the relationship between PA and HRQoL among individuals with diabetes mellitus in South Korea using a comprehensive, nationally representative database (Korea Health Panel Survey [KHPS]), and (ii) to explore depression as a potential mediator of the association between PA and HRQoL.

Methods

Study design.

This study obtained data from the 2020 KHPS dataset. The KHPS was undertaken collaboratively by the National Health Insurance Corporation and the Korea Institute for Health and Social Affairs. The data presented in this study is provided at the request of the author and is not disclosed for privacy purposes, https:// www.khp.re.kr:444/ (accessed on August 10, 2023). It is a nationwide public database comprised of households and their members. Each household and member is given a unique, confidential identification number protected by the data-gathering system. The data collection system and databases do not link the identification numbers to personal information to protect respondents' confidentiality. Trained interviewers conduct computer-assisted personal interviews with study participants in their homes.

The KHPS was separated into three study types: individual, household, and disease surveys. The household survey included questions about demographic characteristics, household premiums,

living expenses, drug purchases, and private health insurance. The data collection process began with authors downloading and completing the data use agreement. Next, the respondent sent the completed form to KHPS and awaited approval and an email from KHPS. After approval and receiving the data by email, the KHPS extracted the data using the SPSS program. We only utilized open data from the KHPS and excluded any personal identification information. The study sample was derived from respondents who self-reported being diagnosed with diabetes mellitus. These individuals were asked whether they had chronic diabetes, to which they could respond yes or no. The inclusion and exclusion criteria were as follows. In 2020, respondents with diabetes mellitus (*n* = 1,543) were selected from among all KHPS respondents (*N* = 14,844). Then, those who did not report their PA levels (*n* = 1,503) or provide information about their alcohol consumption (*n* = 1,472) were excluded from the analysis. After excluding those with incomplete or missing information, the analysis comprised 1,472 respondents with diabetes mellitus **(Fig 1)**.

Figure 1. Flow diagram displaying the inclusion and exclusion of subject.

Measures

PA

We used the International Physical Activity Questionnaire short form (IPAQ-SF) to assess PA duration and frequency. The respondents were asked two key questions: “Over the past week, how many days did you participate in vigorous or moderate-intensity physical activities that caused slight breathlessness and an elevated heart rate for a minimum of 10 consecutive minutes?” and “On those days when you engaged in vigorous or moderate PA, approximately how many minutes did you typically dedicate to such activities?” The total time spent in PA was then calculated as the average number of days in the previous week where the respondent engaged in at least 20 minutes of vigorous-intensity PA or at least 30 minutes of moderate-intensity PA [13].

We also assessed the respondents' regular exercise and walking activity. Regular exercise was evaluated using the question, "Over the past year, have you exercised regularly?" The possible answers were "no exercise" and "regular exercise." Regardless of the type or degree of intensity, the categorization of PA into regular and irregular groups was based only on the frequency of engagement in PA. Finally, walking activity was evaluated using the question, "How many days did you walk for at least ten minutes at a time throughout the last week?" The walking variables were categorized into three groups based on the seven-category answer options: "none," "≤3 days/week," and ">3 days/week."

Depressive symptoms

Depressive symptoms were assessed in the KHPS through the question, "In the past year, have you experienced sadness or unhappiness for more than two weeks that disrupted your daily life?" The possible responses were "yes," indicating the presence of depressive symptoms, and "no," indicating the absence of depressive symptoms.

HRQoL

HRQoL has been assessed in the KHPS using the EuroQol-five dimension (EQ-5D) instrument since 2009 and is presently the subject of investigation. The KHPS examines several HRQoL factors, such as motor skills, daily activities, self-management, pain/discomfort, and anxiety/depression. Each question that described the respondent's current situation was assigned a point value: 1 = no problem, 2 = some problem, and 3 = extra problem. Higher scores indicated worse HRQoL. The EQ-5D index was analyzed based on the South Korean Time Trade-Off Values for EQ-5D health states as follows: EQ-5D index = 1 − (0.05 + 0.096×[Mobility = 2] + 0.418*[Mobility = 3] + 0.046×[Self-Care = 2] + 0.136×[Self-Care = 3] + 0.051×[Usual Activity =

2] + 0.208×[Usual Activity = 3] + 0.037×[Pain/Discomfort = 2] + 0.151×[Pain/Discomfort = 3] + 0.043×[Anxiety/Depression = 2] + 0.158×[Anxiety/Depression = 3] + 0.05×N3, where N3 is an interaction term. A dimension is classified as 1 if it belongs to level 2 or 3 and 0 otherwise. The weighted score was 1 when all five aspects of EQ-5D were rated as 1. A higher EQ-5D score indicates better HRQoL [14].

Covariates

The following variables were included in the analysis: smoking, body mass index (BMI), drinking habits, high stress, anxiety symptoms, suicidal thoughts, age, education level, economic situation, family composition, sex (male and female), and subjective health status. The analysis comprised individuals aged 20–94 years, who were divided into four age groups: 20–29, 30–49, 60–64, and ≥65. Age was examined using the mean and standard deviation (SD). Education level was determined based on the KHPS level, which was then graded using a six-point Likert scale and restructured into dichotomous categories based on the study by Kim et al. as “employed” or “unemployed.”[15]. Family compositions were divided into “living alone” and “living with others” based on the question, “Do you currently live in this household together?” Disability status was dichotomized into disabled and non-disabled. The BMI was calculated from the respondents’ height and weight using the following formula: weight (kg)/height (m^2). According to the Korean Society for the Study of Obesity, respondents were considered obese if they had a BMI of ≥25 kg/m^2.

Smoking status was categorized into three groups: never-smoker, ex-smoker, and current smoker. Following Han et al. (2023), respondents who stated that they had smoked in the last 30 days were categorized as current smokers, and those who have abstained from smoking for one year or more are classified as ex-smokers. Alcohol use was categorized into three groups depending

on the quantity and frequency of drinking: non-drinkers, moderate drinkers, and heavy drinkers. The KHPS question asked respondents to rate the stress they typically encounter daily as 1 (high stress levels), 2 (I often experience high stress levels), 3 (low stress levels), and 4 (no stress). The respondents provided ratings indicating their stress level, with two possible answers: yes (indicating a high stress level) and no (indicating no stress).

Anxiety symptoms were assessed by asking respondents about their experiences with excessive anxiety or worry that interfered with their daily lives for at least six months. The responses indicating the presence of anxiety symptoms were categorized as yes and no. Suicidal thoughts were assessed using the question, “Have you ever thought about dying in the past year?” and responses were categorized as yes and no. Finally, respondents’ subjective health status was classified as “poor” or “fair/good” based on their general health descriptions.

Statistical analysis

The data were analyzed using the SPSS software (version 25.0; IBM, Armonk, NY, USA) and JAMOVI (version 2.5.3). Categorical data are described using frequencies (percentages), and continuous data are described using means (SDs). Simple linear regression analyses based on the HRQoL measure were conducted to obtain unadjusted variable values. Then, multiple linear regression analyses were performed using the entered model to adjust each variable for HRQoL. Mediation analyses were conducted using the MEDMOD package in JAMOVI to assess direct and indirect effects. These moderated mediation models included regular exercise, walking, and PA as independent variables, depressive symptoms as mediators, and HRQoL as the dependent variable. A two-tailed *p*-value of <0.05 was considered statistically significant for all analyses.

Ethical statements

This study was conducted in accordance with the Declaration of Helsinki. The Korean Health Panel data used in this study is statistical data designated by the government pursuant to Article 18 of the Statistics Act (Approval No. 920012). The contents of the questionnaire and survey results are only used for statistical purposes in establishing national policy. This study was exempt from ethical review and approval because only de-identified data was providesd and used in accordance with the Personal Information Protection Act and Statistics Act to prevent individuals from being inferred from survey data. In addition, all personal information cannot be used to identify individuals and is fully protected in accordance with Articles 33 and 34 of the Statistics Act. Before participating in the KHPS, respondents were asked to read and sign an agreement form, which consented to use their data in future scientific research. All participants in this study received sufficient explanation about how to use the questionnaire, provided written consent, and then filled out the questionnaire. This study does not include minors.

Results

Participants' characteristics

The study sample exhibited a relatively balanced sex distribution, comprising 49% males and 51% females. Most respondents were aged 60 to 64 years, lived with others, were employed, and had no disabilities. Regarding PA levels, an equal proportion of respondents engaged in regular exercise and did not (50% each). Additionally, more than half of the respondents (53.5%) reported walking for at least three days per week. Light PA (40.6%) was more common than moderate and vigorous PA. Of the 1,472 respondents in the study sample, 27.3% experienced high stress levels, 11.8% exhibited depression symptoms, 8.1% reported anxiety, and 7.4% had suicidal thoughts.

Furthermore, most respondents (44.4%) rated their subjective health status as somewhat. Their mean HRQoL score was 0.878 **(Table 1).**

Figure 2 illustrates the percentage of respondents who reported issues in the five EQ-5D dimensions, disaggregated by sex. More men than women reported having no problems in each dimension: Mobility (37.8% vs. 28%), Self-Care (44.8% vs. 43.5%), Usual Activities (41% vs. 34.9%), Pain/Discomfort (29.5% vs. 17.8%), and Anxiety/Depression (42% vs. 39.3%). The most common concern for both sexes was the Pain/Discomfort dimension, while the least common was the Self-Care dimension **(Fig 2)**.

Figure 2. Distribution of EuroQol 5-dimensions by sex.

The factors that were initially considered to have an impact on HRQoL, such as sex, age >65 years, smoking status, drinking status, high stress, and anxiety symptoms, were found to not be significant after adjustment. In contrast, economic status, disability, and subjective health status consistently influenced HRQoL before and after adjustment ($p < 0.001$). The primary variable in this study was HRQoL, which showed a negative association with depression ($B = -0.051$, 95% confidence interval [CI] = −0.026–−0.076) and positive associations with regular exercise, the three types of walking status, and PA ($p < 0.001$) (Table 2).

Tables 2 and 3 reveal several significant associations and mediating effects. HRQoL exhibited a negative relationship with depression and positive associations with regular exercise, varying levels of walking activity, and different PA intensities ($p < 0.001$). The associations between HRQoL and regular exercise, walking, and PA were found to be mediated by depression. Specifically, the indirect effect of depression accounted for 33.82% ($B = -0.003$, $p < 0.001$), 46.61% ($B = 0.002$, $p < 0.05$), and 13.86% ($B = 0.003$, $p < 0.05$) of the effect of regular exercise, PA, and walking on HRQoL, respectively. Figure 3 shows the interrelationships among PA, walking, regular exercise, depression, and HRQoL. These results indicate that depressive symptoms partially

mediate the relationships between PA levels, active participation in regular exercise, walking frequency, and HRQoL **(Fig 3)**.

Figure 3. Estimation of direct effects of physical activity, walking, regular exercise on HRQoL through depression.

Discussion

Our study was the first to investigate the relationship between PA and HRQoL among individuals with diabetes mellitus in South Korea. Utilizing a large, nationally representative sample from the KHPS, our study provides novel insights into this association within the South Korean diabetic population. Our findings diverge from those of previous epidemiological studies, which have primarily focused on examining depression as a mediator between PA and HRQoL in elderly populations. Differences in sociodemographic characteristics and medical profiles between the elderly cohorts of past studies and the respondents in our study limit the direct comparison of results. Our findings are also expected to contribute to developing targeted interventions to improve HRQoL among individuals with diabetes in South Korea. By elucidating the intricate relationships between PA, depression, and HRQoL within this population, our study provides valuable insights to inform the design and implementation of tailored strategies to enhance overall well-being in the diabetic population.

In our study, most individuals with diabetes performed regular exercise and light PA. A previous countrywide cross-sectional study in England showed that patients with diabetes exhibited no regular exercise, poor eating habits, high smoking, high BMI, high comorbidities, high stress levels, and poor HRQoL [3]. In contrast, individuals with diabetes in South Korea tended to smoke

less, were non-obese, had lower perceived stress levels, and had an average total HRQoL score of 0.8 out of a maximum of 1, indicating no significant problems with their HRQoL.

Our study found that men had better HRQoL than women, consistent with other studies that found men with type 2 diabetes had a slightly greater overall HRQOL than women with type 2 diabetes [16,17], who reported more diabetes-related worries and less ability. Furthermore, HRQoL can be influenced by various factors, including psychosocial challenges (e.g., more significant diabetes-related distress and body image issues), biological factors (e.g., higher risk of certain complications), barriers to accessing quality diabetes care, and socioeconomic disparities (e.g., lower incomes and education levels) [18,19].

Our findings align with previous research, indicating that greater PA and regular exercise are associated with lower risks and fewer symptoms of depression. This negative correlation between PA, exercise, and depressive symptoms is well-established in the literature. For example, a large-scale, cross-sectional, population-based study conducted across 36 low- and middle-income countries revealed a significantly higher prevalence of low PA among individuals with depression than those without depression (26.0% vs. 15.8%, $p < 0.0001$) [20]. Furthermore, systematic reviews and meta-analyses assessing the effects of PA on depression in individuals with type 2 diabetes mellitus have consistently demonstrated the effectiveness of PA in reducing the severity of depressive symptoms (standardized mean difference = −0.57, $p < 0.05$). These findings underscore the importance of promoting PA and regular exercise as potential strategies for mitigating the risk and alleviating the symptoms of depression, particularly among individuals with chronic conditions such as diabetes mellitus. By incorporating regular PA into their lifestyles, they may experience improvements in their mental well-being and overall HRQoL.

PA has been found to alleviate depressive symptoms through various physiological pathways. One key mechanism involves the increased production of neurotrophic factors, such as

brain-derived neurotrophic factor (BDNF), which promotes neuroplasticity and the generation of new neurons (neurogenesis) in the brain. These processes improve depressive symptoms by enhancing neural connectivity and facilitating the growth of new neural pathways [21]. Moreover, regular exercise plays a vital role in regulating the levels and activity of neurotransmitters such as serotonin, norepinephrine, and dopamine, which are crucial for maintaining a balanced mood state. By modulating these neurotransmitter systems, PA can help restore the proper functioning of mood regulation pathways in the brain [21]. Another important mechanism through which PA combats depression is normalizing the dysregulated activity of the hypothalamic-pituitary-adrenal axis. This axis controls how the body reacts to stress, and its dysregulation has been associated with the development of depressive disorders. The release of mood-enhancing hormones and growth factors such as endorphins, vascular endothelial growth factor (VEGF), insulin-like growth factor 1 (IGF1), oxytocin, and arginine vasopressin further contribute to the antidepressant effects of exercise [22,23]. However, several studies have found this relationship to be bidirectional [24,25].

Consistent with previous research, our study found that depression can influence HRQoL. Patients suffering from both depression and diabetes frequently reported greater difficulties with mobility and self-care activities and increased pain and discomfort than those with diabetes alone [26]. The effects of depression on HRQoL domains are likely due to the added burden of managing a chronic illness such as diabetes, the physiological mechanisms underlying both conditions, and the reduced motivation and ability to care for oneself properly. Addressing mental health should be a crucial element of comprehensive diabetes care to improve overall well-being in this population.

Our study showed that regular exercise, walking activity, and different types of PA are also positively associated with HRQoL. This finding is consistent with a previous study by Abonie et al. (202[illegible]), which explored the relationship between PA patterns and QoL among individuals with type 2 diabetes mellitus in Ghana. They revealed that PA, particularly walking and vigorous-

intensity activities, was associated with better QoL outcomes. Another study also found that individuals who did PA and regularly walked showed significantly better general health, vitality, and social functioning, which are part of HRQoL [28].

Depression significantly mediated the relationship between HRQoL and PA, indicating that PA influences HQoL directly and that depression acts as an indirect mediator. Patients with diabetes are at significantly greater risk of developing depression, with prevalences from 18% to 21% [29,30]. While the mediation model accounted for a substantial portion of the variance in HRQoL, additional mediating factors may exist beyond depression. Depression may explain only one aspect of the relationship between PA and HRQoL. Further research is warranted to explore the potential role of other mediating variables that could contribute to this association.

Our findings emphasize the mediating effect of depression on the relationship between PA and HRQoL. They highlight the importance of mental health concerns in individuals with diabetes as part of a comprehensive approach to improving their overall well-being. They also emphasize the significance of developing and implementing targeted programs that assist in preventing or reducing depression among individuals with diabetes. In addition to promoting PA, healthcare providers and policymakers can work towards enhancing HRQoL outcomes for individuals with diabetes by addressing the mental health challenges faced by this patient population.

Our study had several limitations that should be considered. First and foremost, its reliance on self-report scales for measurement may introduce a potential for information bias since respondents' answers may be subject to recall errors or influenced by social desirability. Second, its cross-sectional research design precludes establishing a causal relationship between PA and HRQoL. Despite these limitations, our study had several strengths, including a large sample size of 1,472 individuals with diabetes. Furthermore, its analysis considered important confounding factors, enhancing the validity of the findings. Our results highlight the significance of considering

mental health factors when examining the influences of PA on HRQoL outcomes in this patient population. Furthermore, they highlight the importance of developing comprehensive interventions that address both PA promotion and depression management, with the ultimate goal of optimizing the well-being of individuals with diabetes.

Conclusions

In conclusion, HRQoL was significantly and positively correlated with PA, regular exercise, and differing degrees of walking activity. In contrast, HRQoL was significantly and negatively correlated with depression. The moderating effect of depression on the relationship between PA and HRQoL was confirmed in individuals with diabetes. It is imperative to conduct depression screening and symptom management in this population, which will allow the implementation of strategies that effectively enhance HRQoL via PA interventions.

List of Abbreviations

BMI	Body mass index
EQ-5D	EuroQol-five dimension
HRQoL	Health-related quality of life
KHPS	Korea Health Panel Survey
PA	Physical activity

Author Contributions

K-SH designed research projects and methodologies, reviewed, and revised the final version of the manuscript. DP performed data analysis, interpreted the data, and drafted the manuscript. All authors have read and agreed to the published version of the manuscript.

Funding: None.

Data Availability Statement: The data in this study use the results of a survey conducted by the Korean Health Panel to provide data that has been approved by the National Statistics Office (approval number 920012). The contents of the questionnaire and survey results are only used for statistical purposes in establishing national policy. The data presented in this study were provided by the Korean Health Panel Institute at the request of the corresponding author, available at https://www.khp.re.kr:444/ (accessed August 10, 2023), and were not publicly available for privacy reasons. When publishing research results to use Korean Health Panel data, researchers clearly explain the data management organization, name and version of the data, and adhere to the conditions that the data will only be used for research purposes and will not be loaned or transferred to others. Data was provided by the Korean Medical Panel data manager after sending an email (khp@kihasa.re.kr) with a data use consent form with the requirement of complying with established regulations.

Conflicts of Interest: The authors declare no conflict of interest.

References

1. Oyewole OO, Odusan O, Ale AO, Sodeinde KJ. Effect of short-term glycemic control and physical activity on health-related quality of life among type 2 diabetes receiving care in a tertiary health facility in Ogun State, Nigeria: a cross-sectional study. Pan Afr Med J.

2023;44. doi: 10.11604/pamj.2023.44.47.35680

2. Xu H, Tang L, Hu Z, Gao F, Yang Y, Qin L, et al. Association between physical activity and health-related quality of life in elderly individuals with pre-diabetes in rural Hunan Province, China: a cross-sectional study. BMJ Open. 2018 Apr 12;8(4):e019836. doi: 10.1136/bmjopen-2017-019836

3. Mortensen SR, Kristensen PL, Grøntved A, Ried-Larsen M, Lau C, Skou ST. Determinants of physical activity among 6856 individuals with diabetes: a nationwide cross-sectional study. BMJ Open Diabetes Res Care. 2022 Aug 17;10(4):e002935. doi: 10.1136/bmjdrc-2022-002935

4. Eastwood S V, Mathur R, Atkinson M, Brophy S, Sudlow C, Flaig R, et al. Algorithms for the Capture and Adjudication of Prevalent and Incident Diabetes in UK Biobank. Herder C, editor. PLoS One [Internet]. 2016 Sep 15;11(9):e0162388. doi:10.1371/journal.pone.0162388

5. Young KG, McGovern AP, Barroso I, Hattersley AT, Jones AG, Shields BM, et al. The impact of population-level HbA1c screening on reducing diabetes diagnostic delay in middle-aged adults: a UK Biobank analysis. Diabetologia. 2023 Feb 22;66(2):300–9. doi: 10.1007/s00125-022-05824-0

6. Martins KAKF, Mascarenhas LPG, Morandini M, Cat MNL, Pereira RM, Carvalho JR de, et al. Health-related quality of life in a cohort of youths with type 1 diabetes. Rev Assoc Med Bras. 2018 Nov;64(11):1038–44. doi: 10.1590/1806-9282.64.11.1038

7. Sahrakorpi N, Engberg E, Stach-Lempinen B, Tammelin TH, Kulmala J, Roine RP, et al. Physical activity and health-related quality of life among high-risk women for type 2 diabetes in the early years after pregnancy. BMC Womens Health. 2022 Mar 21;22(1):84. doi: 10.1186/s12905-022-01664-7

8. Huang YC, Zuñiga J, García A. Illness perceptions as a mediator between emotional distress and management self-efficacy among Chinese Americans with type 2 diabetes. Ethn Health [Internet]. 2022 Apr 3;27(3):672–86. doi: 10.1080/13557858.2020.1817339
9. Mishra SR, Sharma A, Bhandari PM, Bhochhibhoya S, Thapa K. Depression and Health-Related Quality of Life among Patients with Type 2 Diabetes Mellitus: A Cross-Sectional Study in Nepal. PLoS One. 2015 Nov 23;10(11):e0141385. doi: 10.1371/journal.pone.0141385
10. Kim DJ. Effects of Physical Activity on Depression in Adults with Diabetes. Osong Public Heal Res Perspect. 2018 Aug 31;9(4):143–9. doi: 10.24171/j.phrp.2018.9.4.02
11. Laird E, Herring MP, Carson BP, Woods CB, Walsh C, Kenny RA, et al. Physical activity for depression among the chronically Ill: Results from older diabetics in the Irish longitudinal study on ageing. Psychiatry Res. 2023 Aug;326:115274. doi : 10.1016/j.psychres.2023.115274
12. Egede LE, Hernández-Tejada MA. Effect of comorbid depression on quality of life in adults with Type 2 diabetes. Expert Rev Pharmacoecon Outcomes Res. 2013 Feb 9;13(1):83–91. doi : 10.1586/erp.12.86
13. Han J, Ju YJ, Lee SY. Physical activity, sedentary behavior, and cardiovascular disease risk in Korea: a trajectory analysis. Epidemiol Health. 2023 Feb 22;45:e2023028. doi : 10.4178/epih.e2023028. doi : 10.4178/epih.e2023028
14. Lee YK, Nam HS, Chuang LH, Kim KY, Yang HK, Kwon IS, et al. South Korean Time Trade-Off Values for EQ-5D Health States: Modeling with Observed Values for 101 Health States. Value Heal. 2009 Nov;12(8):1187–93. doi : 10.1111/j.1524-4733.2009.00579.x
15. Jung B, Kim J, Ha IH, Lee J. Factors affecting utilisation of traditional Korean medical services by privately insured persons: a retrospective study using Korean Health Panel

Survey (KHPS). BMJ Open. 2020 Jan;10(1):e033159. doi: 10.1136/bmjopen-2019-033159

16. Undén AL, Elofsson S, Andréasson A, Hillered E, Eriksson I, Brismar K. Gender differences in self-rated health, quality of life, quality of care, and metabolic control in patients with diabetes. Gend Med. 2008 Jun;5(2):162–80. doi : 10.1016/j.genm.2008.05.003

17. Krzemińska S, Bąk E, Polanská A, Hašová K, Laurinc M, Zrubcová D, et al. Does gender affect health-related quality of life in patients with type 2 diabetes (ADDQoL) in Central European countries? Ann Agric Environ Med. 2023 Jun 26;30(2):296–305. doi : 10.26444/aaem/161584

18. Campbell L V. What do People with Diabetes Distress want from their Diabetes Care Providers: are there Gender Differences? Curr Res Diabetes \& Obes J. 2019;9(3). doi: 10.19080/crdoj.2019.09.555762. doi : 10.19080/crdoj.2019.09.555762

19. Ding C, Zhang J, Lau ESH, Luk AOY, So WY, Ma RCW, et al. Gender differences in the associations between insomnia and glycemic control in patients with type 2 diabetes: A cross-sectional study. Sleep. 2019;42(4). doi : 10.1093/sleep/zsz014

20. Stubbs B, Koyanagi A, Schuch FB, Firth J, Rosenbaum S, Veronese N, et al. Physical activity and depression: a large cross-sectional, population-based study across 36 low- and middle-income countries. Acta Psychiatr Scand. 2016 Dec 5;134(6):546–56. doi : 10.1111/acps.12654

21. Kandola A, Ashdown-Franks G, Hendrikse J, Sabiston CM, Stubbs B. Physical activity and depression: Towards understanding the antidepressant mechanisms of physical activity. Neurosci Biobehav Rev. 2019 Dec;107:525–39. doi : 10.1016/j.neubiorev.2019.09.040

22. Bueno-Antequera J, Munguía-Izquierdo D. Exercise and Depressive Disorder. In 2020. p. 271–87. doi : 10.1007/978-981-15-1792-1_18

23. Hu MX, Turner D, Generaal E, Bos D, Ikram MK, Ikram MA, et al. Exercise interventions

for the prevention of depression: a systematic review of meta-analyses. BMC Public Health [Internet]. 2020 Dec 18;20(1):1255. doi : 10.1186/s12889-020-09323-y

24. Buchan MC, Romano I, Butler A, Laxer RE, Patte KA, Leatherdale ST. Bi-directional relationships between physical activity and mental health among a large sample of Canadian youth: a sex-stratified analysis of students in the COMPASS study. Int J Behav Nutr Phys Act. 2021 Oct 9;18(1):132. doi : 10.1186/s12966-021-01201-z

25. Jang S. Temporal and bidirectional association of depression and physical illnesses: Analyzing the pooled data from independently conducted cross-sectional national surveys at three distinct time points. J Psychosom Res. 2024 Apr;179:111614. doi : 10.1016/j.jpsychores.2024.111614

26. Arsh A, Afaq S, Carswell C, Bhatti MM, Ullah I, Siddiqi N. Effectiveness of physical activity in managing co-morbid depression in adults with type 2 diabetes mellitus: A systematic review and meta-analysis. J Affect Disord. 2023 May;329:448–59. doi : 10.1016/j.jad.2023.02.122

27. Abonie US, Ofori - Ampomah AK, Makinyi V, Addo RA, Kumah L. Associations between physical activity patterns and quality of life in persons with type 2 diabetes: A cross sectional study. Kasujja FX, editor. PLoS One. 2023 Aug 30;18(8):e0290825. doi : 10.1371/journal.pone.0290825

28. Marquez DX, Aguiñaga S, Vásquez PM, Conroy DE, Erickson KI, Hillman C, et al. A systematic review of physical activity and quality of life and well-being. Transl Behav Med. 2020 Oct 12;10(5):1098–109. doi : 10.1093/tbm/ibz198

29. Engidaw NA, Wubetu AD, Basha EA. Prevalence of depression and its associated factors among patients with diabetes mellitus at Tirunesh-Beijing general hospital, Addis Ababa, Ethiopia. BMC Public Health. 2020 Dec 22;20(1):266. doi : 10.1186/s12889-020-8360-2

30. Abuhegazy H, Mujairi A, Banah F, Agdi Y, Elkeshishi H, Kamel A, et al. Depression and Associated Risk Factors Among Type 2 Diabetic Patients: A Cross Sectional Study on a Convenience Sample from the Diabetic Center, Khamis Mushait; Saudi Arabia. Neuropsychiatr Dis Treat. 2022 Aug;Volume 18:1975–84. doi : 10.2147/NDT.S374752

 Click here to access/download;Figure;Fig 1.tif

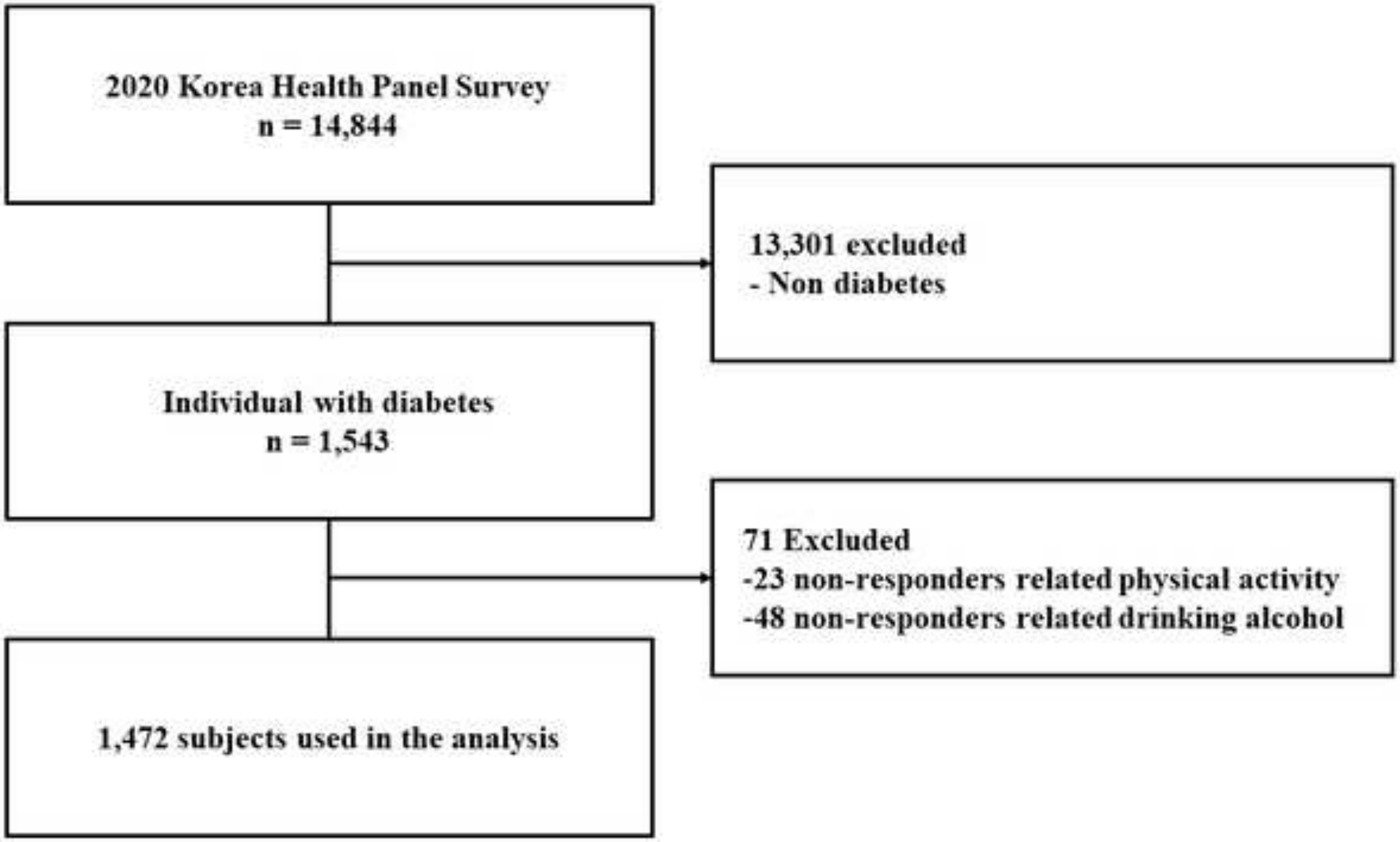

Cover Letter

Click here to access/download;Figure;Fig 2.tif

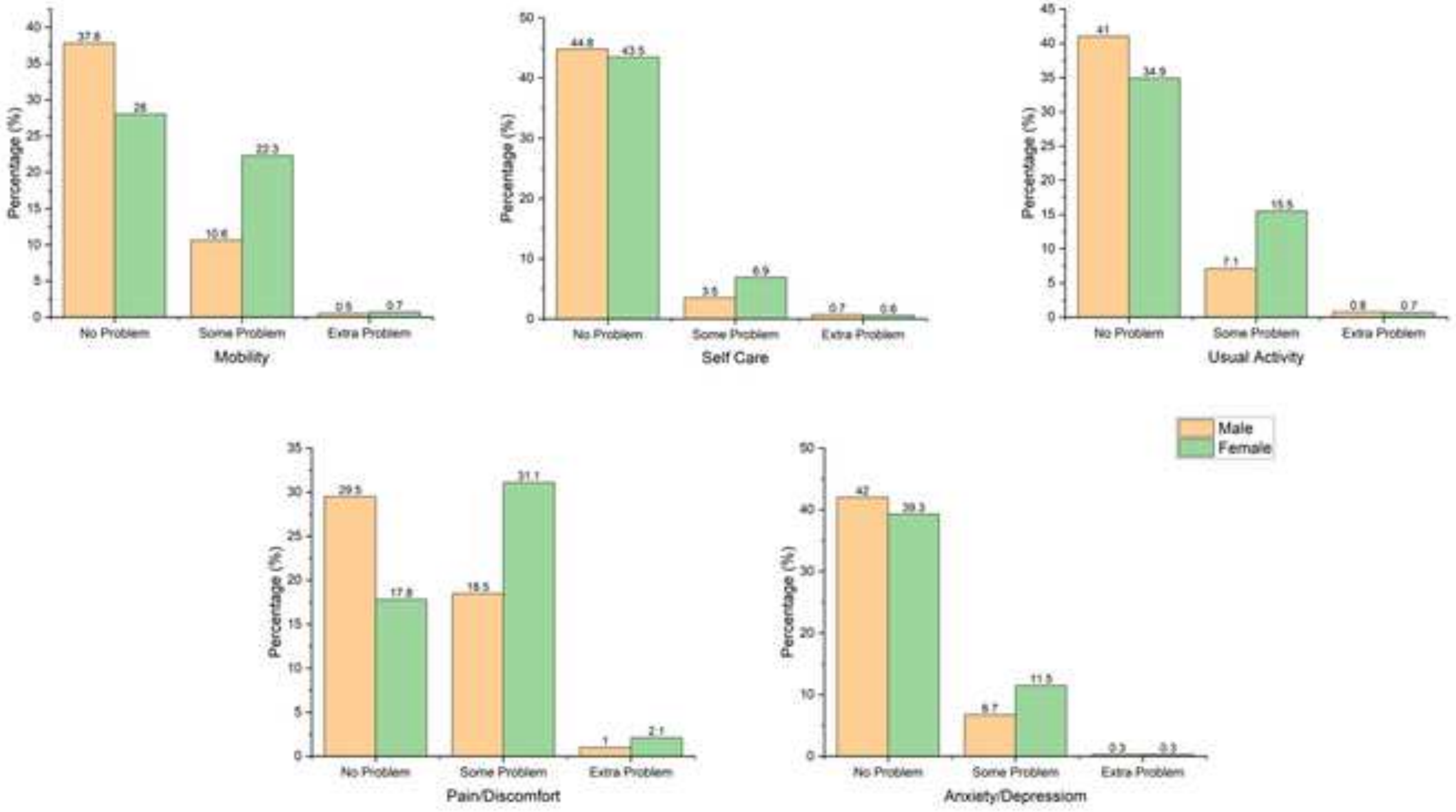

Cover Letter
Click here to access/download;Figure;Fig 3.tif

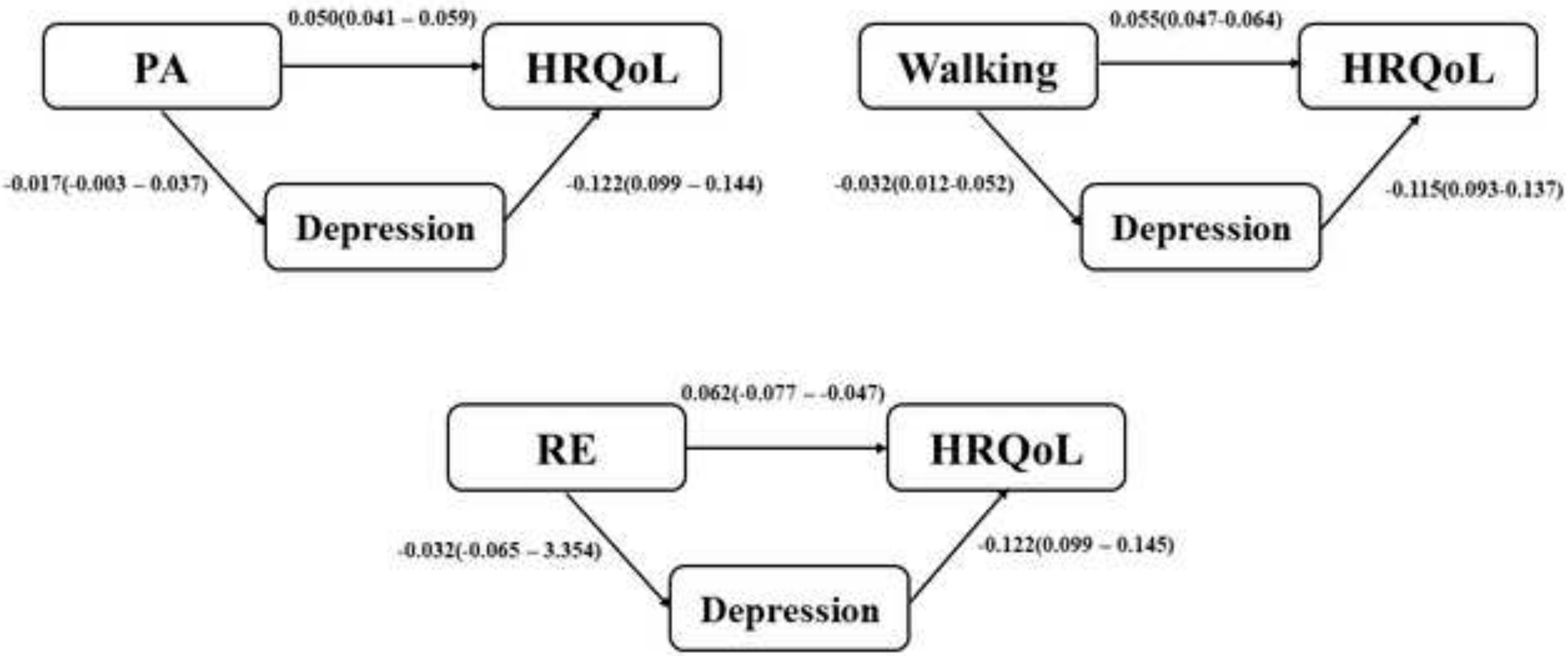

Supplement: Attachment — Submitted filename: PONE-D-24-24159.pdf [file pone.0312774.s001.pdf]
