# Supplementary material for: The mediating role of depression on the link between physical activity and health-related quality of life among people with diabetes: A cross-sectional study
Source: PLoS One. 2024 Dec 5;19(12):e0312774. doi: 10.1371/journal.pone.0312774 (PMC11620351; doi:10.1371/journal.pone.0312774)
Supplement: Attachment — Submitted filename: Reviewer Comments.docx [file pone.0312774.s002.docx]

**Reviewer Comments**

**Introduction**

- Provide reference for line 40 (sentence starting with “However…..”)

**Ethical consideration**

Line 184: check the spelling of ‘providesd’

**Results**

Lines 196-197: The sentence starting with “Regarding PA levels….) must be rephrased to make the meaning clearer
